# Study of *Cis*-regulatory Elements in the Ascidian *Ciona intestinalis*

**DOI:** 10.2174/138920213804999192

**Published:** 2013-03

**Authors:** Steven Q Irvine

**Affiliations:** Department of Biological Sciences, University of Rhode Island, Kingston, RI 02881, USA

**Keywords:** Chordate, tunicate, amphioxus, transgenics, reporter, transcription, promoters, enhancers.

## Abstract

The ascidian (sea squirt) *C. intestinalis* has become an important model organism for the study of *cis-*regulation. This is largely due to the technology that has been developed for assessing *cis-*regulatory activity through the use of transient reporter transgenes introduced into fertilized eggs. This technique allows the rapid and inexpensive testing of endogenous or altered DNA for regulatory activity *in vivo.* This review examines evidence that *C. intestinalis* *cis-*regulatory elements are located more closely to coding regions than in other model organisms. I go on to compare the organization of *cis-*regulatory elements and conserved non-coding sequences in *Ciona*, mammals, and other deuterostomes for three representative *C.intestinalis* genes, *Pax6, FoxAa, *and the* DlxA-B* cluster, along with homologs in the other species. These comparisons point out some of the similarities and differences between *cis-*regulatory elements and their study in the various model organisms. Finally, I provide illustrations of how *C. intestinalis *lends itself to detailed study of the structure of *cis-*regulatory elements, which have led, and promise to continue to lead, to important insights into the fundamentals of transcriptional regulation.

## *CIONA* AS A MODEL FOR *CIS-*REGULATORY STUDY

*Ciona intestinalis* has emerged as an important model organism for the study of genomic regulation in the last 15 years. *C. intestinalis* is an ascidian - a member of the class Urochordata, which along with the cephalochordates are considered basal chordates Fig. (**1**). They have a simple chordate body plan, with a dorsal nervous system, axial skeleton (notochord), ventral heart, and an elaborate pharyngial apparatus. The developmental biology of ascidians has been the subject of detailed study for over 100 years, in part because of their stereotyped cell lineage, which enables experimental study of the relative roles of cytoplasmic inheritance and cell-cell signaling in development [1, 2]. *C. intestinalis* has particular advantages as a laboratory model organism, since it is a common marine organism, and thousands of synchronously, and rapidly, developing fertilized embryos can be obtained easily by *in vitro *fertilization on a daily basis.

The advent of genomics has increased interest in *Ciona* for several reasons [3]. It has a very small genome - approximately 160 Mb. [4], around the size of *Drosophila*, which facilitated the release of whole genome sequences of the congeneric species *C. intestinalis* and *C. savignyii*, by 2003. The genome is compact, with only about 7.5 kb. per gene, making it relatively easy to capture significant portions of transcriptional units in readily cloneable pieces. Significantly, ascidians lack the genome duplications present in vertebrates, so they have only one paralog of many genes that have been duplicated in vertebrates. This lack of duplicate genes reduces the amount of functional overlap and redundancy, simplifying functional genomic study. These characteristics of *Ciona,* among others, have enabled the generation of a host of bioinformatic resources, as summarized in Table **1**. This review concentrates on the progress in *cis-*regulatory study in *C. intestinalis. *It does not cover the large and growing literature on other aspects of genomic research in *Ciona*, such as the elicidation of gene regulatory networks (*eg.* [5-7]).

Perhaps the most important reason for the interest in *Ciona *for *cis-*regulatory study is the development of electroporation as a technique for introducing reporter transgenes into eggs or zygotes [8-11]. This method enables the simple and rapid generation of many transgenic embryos, which develop to the larval stage in around 18 hours. The typical *in vivo *assays performed in *C. intestinalis* are transient - only observed in the generation electroporated with the transgene. For many successful studies, these transient assays have allowed rapid testing of large numbers of experimental *cis*-regulatory element (CRE) variants at low cost [12]. However, some workers have also successfully transmitted transgenes to the germline and reared subsequent generations of transgenic animals. In addition, transposon mediated enhancer trap lines have been reported recently (reviewed in [11]).

While *C. intestinalis *is a chordate, its genome has some significant differences with those of vertebrates. It is very AT rich, with only about 35% GC content. The proportion of repetitive sequence has not been determined precisely, but it is much lower than in many vertebrates, such as humans. It does however, harbor several transposon types [13]. Methylation patterns are also different from vertebrates. Coding sequences of genes are preferentially methylated, but intergenic regions have only half that level of methylation. In vertebrates, on the other hand, the genome is more uniformly methylated, except at promoters, and the overall level of methylation is twice as high as in *C. intestinalis* [14]*.* Like other invertebrates, *C. intestinalis *has a very high level of nucleotide polymorphism, around 1.2% SNPs and indels between alleles. The high allelic variation makes genome assembly a difficult task [4, 15].

**Fig. (1):**
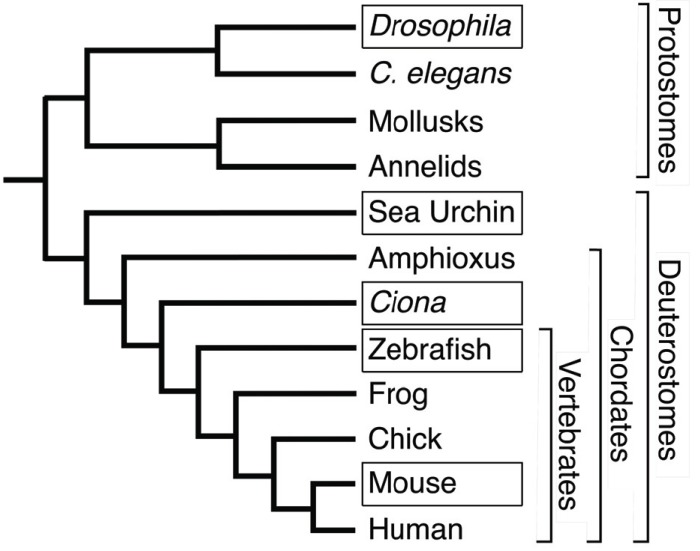
Phylogeny of selected bilaterian animals. Taxa with abundant experimental *cis*-regulatory data are denoted by boxes around the taxon name. Tree topology based on a consensus of recent phylogenetic work eg. [16, 17].

Recent phylogenomic studies have placed the ascidian urochordates as the sister group to the Vertebrata [16, 17] (Fig. **1**). Since *cis-*regulatory experiments are more difficult in other basal chordates, such as amphioxus and lamprey, *Ciona* has become a major model organism for the functional study of the evolution of chordate genomic regulation and the evolution of development [18-20].

## BASIC CHARACTERISTICS OF *CIONA* *CIS-*REGULATORY ELEMENTS

Nearly all reported experimentally confirmed *cis-*regulatory elements (CREs) in *Ciona* are enhancers, which activate transcription in a more or less tissue-specific manner. This is probably due to the fact that other elements, such as insulators or silencers, are more difficult to identify. Table **2** is a compilation of most, if not all, transcription factor and signaling molecule enhancer elements reported to date for *C. intestinalis.* (The table was limited to these catagories in the interest of space, and because these genes have attracted much of the interest in the field.) A 2005 review of ascidian *cis-*regulatory elements listed 11 genes of these types with experimentally determined CREs [21]. As of this writing, the total is up to 63 genes as listed in Table **2**, several with multiple CREs determined. Many other CREs have been reported in *Ciona* in other functional catagories.

**Fig. (2):**
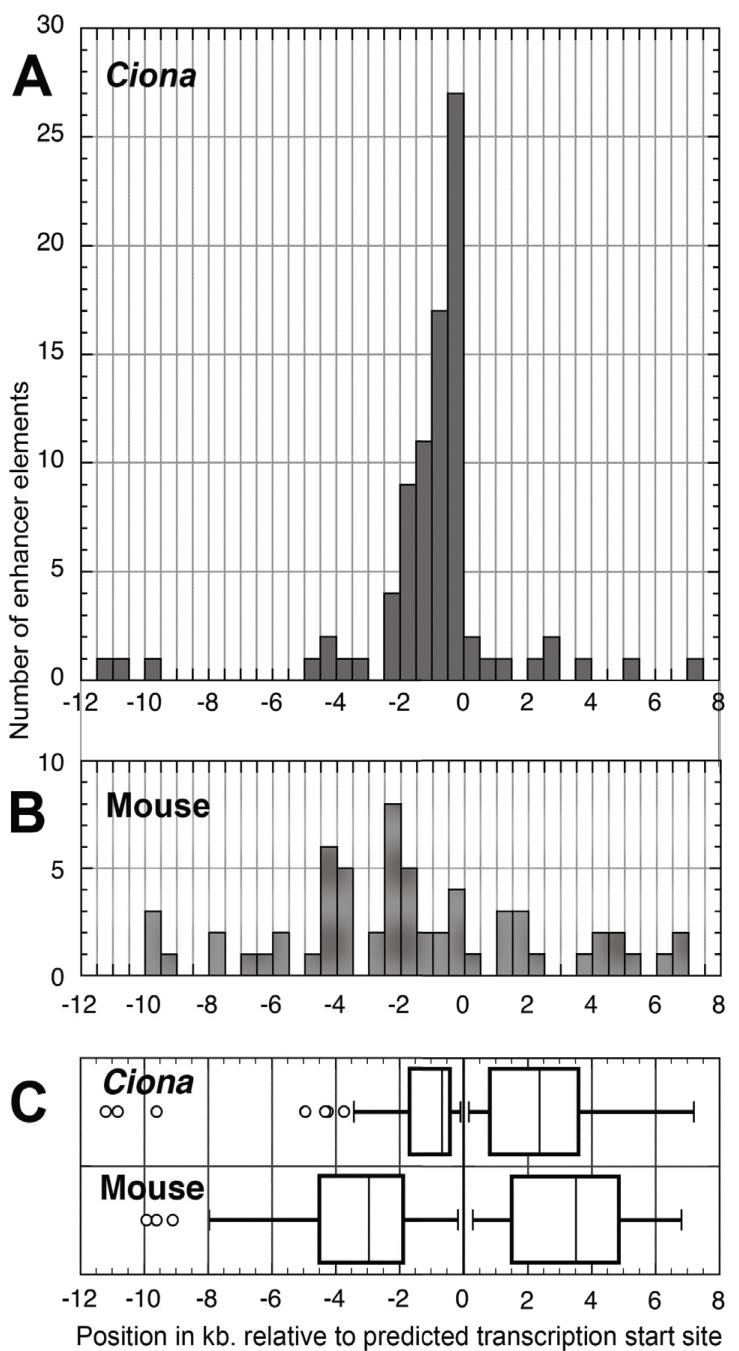
Genomic positions of experimentally localized cis-regulatory elements (CREs) in *C. intestinalis* as compared with those in mouse. **A**) Histogram depicting frequencies of positions of CREs for 62 *C. intestinalis* transcription factor and cell-cell signaling genes. Positions based on 5' or 3' mean distance of experimentally determined *cis*-regulatory elements from the TSS. **B**) Mouse CRE positions for 30 mouse transcription factor and cell-cell signaling genes, depicted as in **A**). Only CREs between -12 and +8 kb relative to the TSS are included. **C**) Distribution of mean positions of CREs compared between *C. intestinalis* and mouse. For *C. intestinalis* and mouse data and references refer to Supplemental Tables **1** and **2**.

The 83 *Ciona* enhancers listed in Table **2** were all found within 12 kb. upstream or 7.5 kb. downstream of their respective estimated transcription start sites (TSSs). Consistent with the compact genome, most enhancers are found within 1.5 kb. upstream of the TSS [22], as shown in Fig. **2A**. Fig. **2B** shows the contrasting condition in the mouse. Out of a random selection of 79 experimentally verified mouse enhancers from 30 different transcription factor and cell-cell signaling genes, those within the total range of the *Ciona* enhancers were much less concentrated close to the TSS, and more likely to be located downstream. In fact, in this sample, mouse enhancers ranged as far as 123 kb downstream and 93 kb upstream. (Refer to Supplemental Material Table **S1** for Ciona enhancer locations, and Table **S2** for the list of mouse genes and enhancer locations).

**Fig. (3):**
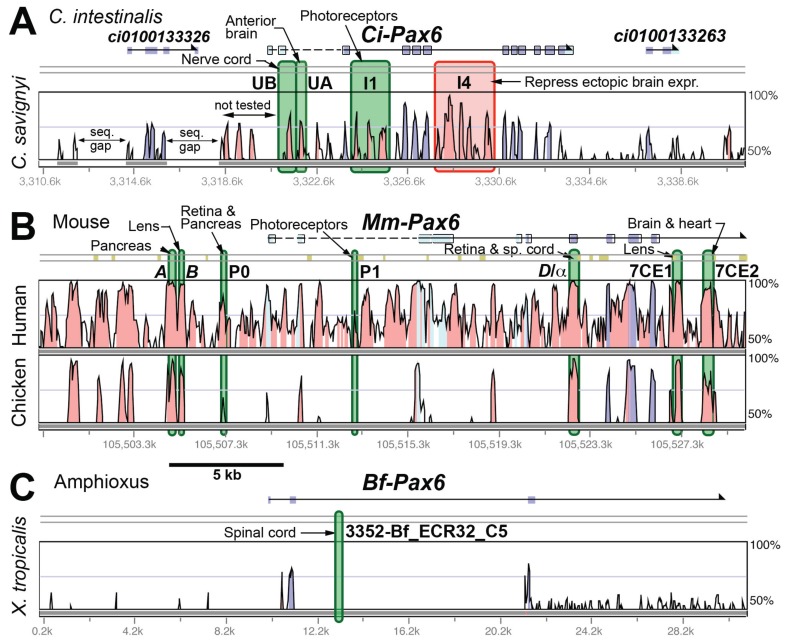
Graphical representations of sequence conservation and CRE locations for Pax6 genes in *C. intestinalis*, mouse and amphioxus at the same genomic scale. Alignments were generated by AVID or LAGAN using the VISTA web application [43]. Experimentally verified CREs are denoted by colored overlays - green for enhancers, red for repressors, blue for CREs that modulate expression in some other way. Regions of expression for each CRE are noted. **A**) Plot of sequence conservation between *C. intestinalis* and *C. savignyi* for the ascidian *Pax6* gene. **B**) Mouse vs. chicken. **C**) Amphioxus *vs. Xenopus.* No more conservation was seen in alignments of amphioxus with human, zebrafish, or lamprey DNA. (CREs: [44-47, 29]).

There is clearly a much greater spread in the locations of mouse CREs as compared with those of *C. intestinalis *(Fig. **2C**). In this sample the median locations of *C. intestinalis *CREs was 630 bp upstream from the TSS and 2.4 kb downstream, while in the mouse the medians were 4.1 kb upstream from the TSS, and 6.3 kb downstream. Because CREs more than 12 kb upstream or 8 kb downstream of the mouse TSSs were ignored in constructing Fig. **3C**, the actual median distance to CREs in the mouse is even further than shown here.

The close position to the TSS of most *C. intestinalis *CREs means that investigators have a good chance of finding major CREs by examining only a few kb upstream of the TSS. However, an important caveat to this axiom is that it is possible that searches for CREs in *C. intestinalis *are biased towards elements proximal to the TSS. The kinds of long-range CRE searches that have been done in mouse, using BAC reporter transgenes, or *Drosophila* using P-element transgenes, for example, have not been done in *C. intestinalis,* so the presence of CREs acting at more than a few kb cannot be ruled out.

This difference in the distances to CREs between the mammal and *C. intestinalis *reflect the extreme compactness of the *Ciona *haploid genome, which is only 160 Mb, as opposed to 2700 Mb in the mouse. However, genome size doesn't necessarily correlate with compactness of CRE architecture, as seen in the large distances to some CREs found in *Drosophila*, which has a similar genome size to *C. intestinalis*****[23]*.* For example, in a very incomplete look at *D. melanogaster* CREs in the Redfly database [24], the most proximal enhancers for the Hox genes *lab* and *Scr* are 1-2 kb 5' of the TSS, and for *Dfd* about 4 kb 5'. However, numerous other enhancers for each gene are found further from the promoter, as much as 40 kb 5' and 25 kb 3' of the TSS for *Scr.* For the fly *Dll* gene, the most proximal enhancer found is about 5 kb 5' of the TSS, with longer range enhancers up to 15 kb 5' and 40 kb 3'. In *C. intestinalis*, on the other hand, the enhancers for *Ci-Hox1* and *Ci-Hox3 *are within 2 kb 5' and 2.5 kb 3' of the TSS, and for *Ci-DllA *and *DllB,* major enhancers are within 0.5 kb 5', without longer range enhancers having been found. Thus, the ascidian may have a constraint on CRE distance that is not present in some other metazoans with small genomes, such as *Drosophila*.

While this apparent constraint on distance of enhancers from transcription start sites may be an artifact of the lack of longer range enhancer searches, if the constraint turns out to be real it may be related to the apparent prevalence of rearrangements in the *Ciona *genome. For example, the Hox and ParaHox genes, which have conserved cluster arrangements in many other animals, are dispersed and rearranged in *C. intestinalis* [25, 26]*.* If the *C. intestinalis *genome is subject to frequent rearrangement, there may be a selective advantage to having enhancers located close to TSSs, since there would be a lower probability of a rearrangement event between the enhancer and basal promoter that might render the enhancer non-functional. In this scenario, enhancers located more distally would be lost to purifying selection if rearranged away from their appropriate basal promoter.

## CONSERVED SEQUENCES AND *CIS-*REGULATORY MODULES

Another advantage of *C. intestinalis* for CRE study is the fortuitous genetic distance to its congener *C. savignyi*, also sequenced, which allows for reliable prediction of CRE positions by finding conserved non-coding elements (CNEs) in alignments of the genomes [27]. Even though *C. savignyi* and *C. intestinalis* are capable of producing viable hybrid offspring by *in-vitro *fertilization, they exhibit very low similarity in most non-coding DNA sequences. Genomic comparisons indicate that the genetic distance between the two congeneric ascidians is in the range of human-chicken or human-frog [27, 28]. The fact that the two ascidians can be hybridized suggests that the coding and non-coding DNA sequences that are highly conserved represent genuine functional sequences. This observation has been borne out by many investigations, and allows for reliable prediction of putative CREs for experimental verification.

**Fig. (4):**
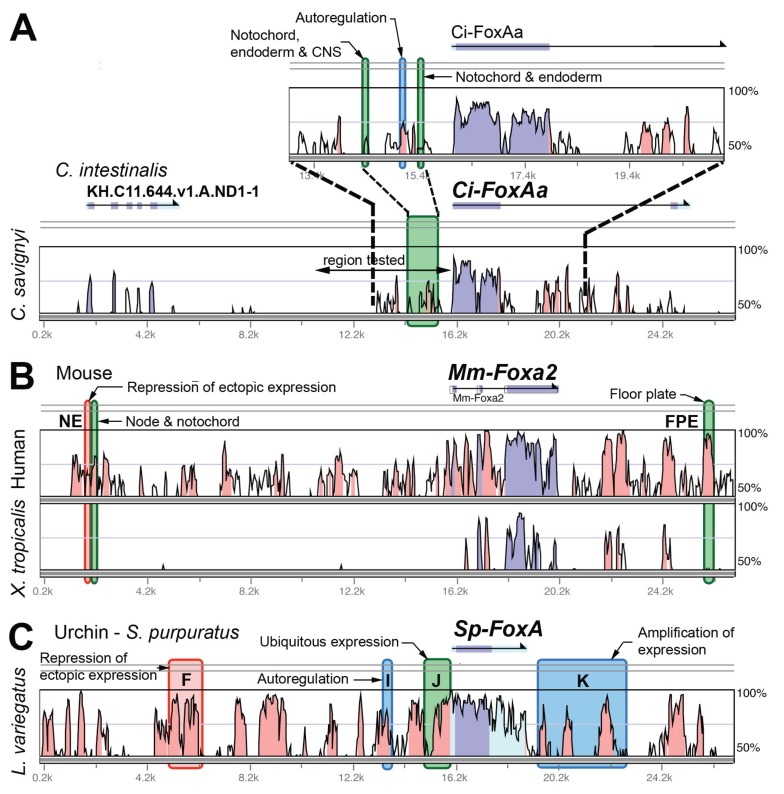
Graphical representations of sequence conservation and CRE locations for FoxA genes in *C. intestinalis,* mouse and sea urchin. Plots constructed as in Fig. **3**. While these genes may not be strictly orthologous, they belong to the same gene subfamily (FoxA. **A**) Plot of sequence conservation between *C. intestinalis* and *C. savignyi* for the ascidian *FoxAa* (a.k.a. *forkhead*) gene. The region proximal to the TSS is shown enlarged. **B**) Mouse *Foxa2 vs.* human and chicken genomes. Note that there are extensive gaps in the mouse *vs.* chicken alignment. **C**) *S. purpuratus vs. L. variegatus FoxA* (two sea urchins). (CREs: [48-50, 30]).

**Fig. (5):**
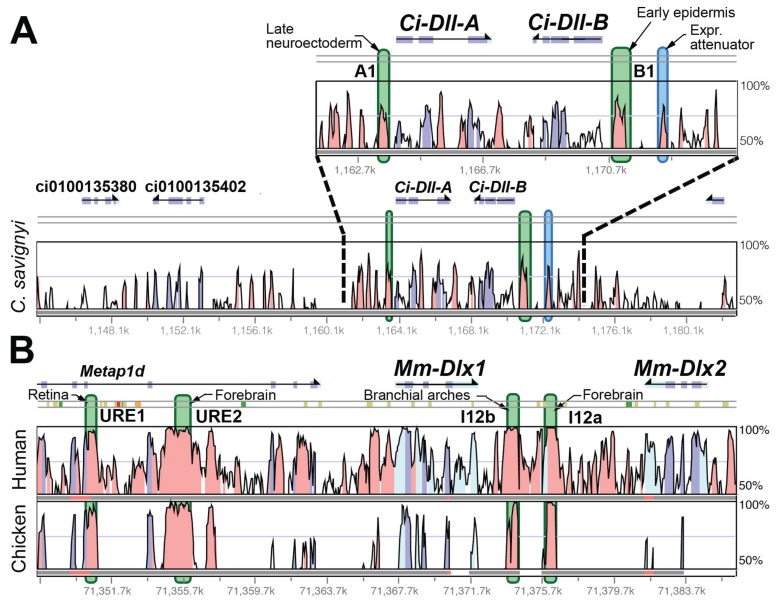
Graphical representations of sequence conservation and CRE locations for *Dlx* cluster genes in *C. intestinalis* and mouse. Plots constructed as in Fig. **3**. The mouse *Dlx1-2* cluster is used in comparison with the *C. intestinalis DllA-B* cluster, since there are more CRE data available for that cluster, although the orthology of the *C. intestinalis* cluster with respect to the three mouse *Dlx* clusters has not been able to be determined by phylogenetic means [31]. **A**) *C. intestinalis Pax6 vs. C. savignyi genome*. The region proximal to the TSSs is shown enlarged. **B**) Mouse *Dlx1-2* cluster *vs.* chicken. (CREs: [37, 51, 52]).

In order to visualize the relationship between CNEs and CREs, Figs. **3-5** map experimentally determined CRE locations onto same-scale plots of sequence conservation for 3 transcription factor genes in *C. intestinalis*, mouse, and certain other taxa. These comparisons were chosen because of the availability of published experimental CRE data along with complete genome sequences. Each comparison provides different insights into CRE characteristics.

### Case 1 - Pax6

In Fig. **3**, data for Pax6 are plotted. Here an alignment for amphioxus *vs.* frog is also included, as a CRE has been reported for this other invertebrate chordate. In the alignment of *C. intestinalis vs. C. savignyi *(Fig. **3A**), each of the four CNEs tested by reporter gene assay had some *cis*-regulatory activity, although the I1 and I4 regions were not dissected in enough detail to exclude the possibility that some individual CNE peaks within those regions are not involved in *cis-*regulation. The mouse *Pax6* gene is clearly orthologous to *Pax6* in *C. intestinalis.* The alignment shown (Fig. **3B**) compares the mouse genome with that of chicken, as the chicken-mouse overall genetic distance is similar to *C. intestinalis-C. savignyi.* More individual CREs have been found in *Mm-Pax6,* than in *Ci-Pax6,* as would be expected given the much greater anatomical complexity of the mammal. (In addition, long-range downstream CREs have been found in the mouse, which are not shown here.) Interestingly, although 5 of the 7 CREs identified map to mouse-chicken CNEs, 2 of the CREs (P0 and P1) do not. In fact, even in the mouse-human comparison, these CREs correspond to regions much less conserved than several others in this region that were not found to have CRE activity. Included in this catagory are 3 upstream and two intronic CNEs conserved with chicken. An unusual case of an active CRE is the "D/α(" element, which is coincident with one of the protein coding exons.

In the case of amphioxus, a survey of CNEs found in an exhaustive and sensitive search [29] located a short (48 bp) CNE in the *Bf-Pax6 *gene Fig. (**3C**). The less sensitive AVID or LAGAN alignment programs implemented in VISTA, and used for the Fig. (**3-5**) plots, failed to show an amphioxus CNE shared with frog, lamprey, zebrafish or human genomes at the empirically identified CRE. For amphioxus, either alignment algorithms more sophisticated than those commonly used, or a more closely related comparison genome, will be required to discover more CREs using sequence analysis.

### Case 2 - FoxA

Empirical data on FoxA CREs are available for *C. intestinalis, *mouse, and sea urchin. In the case of *C. intestinalis*, CREs were carefully mapped on a VISTA plot of CNEs. This mapping shows that 3 of the experimentally identified CREs correspond with CNEs, but two significant CREs do not (the notochord, endoderm, and CNS enhancers in Fig. **4A**). In fact, two CNEs fall just adjacent to CREs. For the mouse *Foxa2* gene 2 of 3 CREs coincide with CNEs in the mouse-human comparison. However, once again there are equally or more conserved sequence elements in the mouse-human comparison that failed to show CRE activity in the published studies. As for a more distant comparison, most of the mouse *Foxa2* locus fails to align with the chicken genome, so the comparison here is with the frog, *X. tropicalis*. None of the 3 identified CREs show up as CNEs in the frog alignment.

A study has also been published of the sea urchin *Strogylocentrotus purpuratus FoxA* gene, which revealed 4 CREs (Fig. **4C**). All 4 correspond with CNEs showing up in an alignment with the urchin *Lytechinus variegatus*, the species the study authors used for comparison [30]. While 98 kb of upstream and 50 kb of downstream sequence was tested for CRE activity, the acting CREs were found within 12 kb upstream and 7 kb downstream of the TSS. Within the 26 kb of sequence shown in Fig. (**4C**), on the other hand, only about half of the CNEs correspond with CREs that show up in the reporter assays. Interestingly, from the work published on these gene loci to date, it appears that the complexity in terms of numbers of CREs regulating each gene is similar in the three taxa, even though anatomical complexity varies widely from *C. intestinalis* to urchin to mouse, and the genes are expressed both early and late in development.

### Case 3 - Dlx Clusters

Both vertebrates and *C. intestinalis* have Dlx homeobox genes arranged in convergently transcribed 2-gene clusters, which are linked to Hox clusters on the same chromosomes [31]. Mammals have 3 such clusters - the relationships of which to the single *C. intestinalis* cluster have not been able to be robustly determined. Because the most CRE data are available for the mouse *Dlx1-2 *cluster, that locus has been used here (Fig. **5**) for comparison. For *C. intestinalis* the major enhancer elements are located proximal to the respective TSSs and correspond with the largest regions of conserved sequence with *C. savignyi* (Fig. **5A**). For *Ci-DllB,* additional upstream sequence in which four CNEs are located was tested, only one of which had an effect (attenuation) on expression.

For the mouse *Dlx1-2* cluster, the most broadly acting CREs are located in the large intergenic region. Other CREs activating *Mm-Dlx1* (URE1 and 2) have been found far upstream in the introns of the *Metap1d* gene model. The number of CREs found to date, 3 in *C. intestinalis* and 4 in mouse, suggest a comparable level of *cis*-regulatory complexity. However, it is possible that more mouse CREs would be found with more extensive searching, and that the individual mouse *cis*-regulatory modules have more internal complexity than those in the ascidian.

For the Dlx clusters the *C. intestinalis-C. savignyi* and mouse-chicken genomic comparisons do a good job of correlating CNEs with CREs. Once again though, several CNEs are found that do not correspond with the CREs uncovered in reporter gene assays.

### Correspondance Between Conserved Non-Coding Elements and cis-regulatory Elements

As found by Johnson *et al.* (2004) [27], in many cases verified *C. intestinalis* CREs correspond to non-coding sequences conserved with *C. savignyi.* (In the present analysis at >70% identity over 75 bp scanned with a 75 bp window in VISTA.) However, even in the small sample of 3 genes examined here, one case - *Ci-FoxAa* - has limited conserved sequence showing up in the commonly used VISTA analysis. Here, a more sensitive conservation-finding method might have located more of the discovered CREs. On the other hand, it may be the case that there is too much rearrangement or reorganization of the CREs since the split of *C. intestinalis* and *C. savignyi* to be able to use sequence analysis as a CRE finding tool. It is likely that this would be the case for a significant minority of both individual CREs, or the complement of CREs for particular genes.

Interestingly, the mouse *Foxa2* gene exhibits a somewhat similar situation to that of *Ci-FoxAa.* In the intermammalian comparison, there are many CNEs with the same or greater degree of conservation to the few that have been empirically shown to have CRE activity, while no sequence conservation with the phylogenetically closest non-mammalian sequenced genome, *X. tropicalis,* was found. In other words, for this gene the consensus of mammalian alignments have too much sequence conservation to efficiently identify CREs, but not enough sequence conservation to the next closest taxon.

Alternative approaches for identifying CREs rely on various ways of searching for shared non-coding motifs, in, for example, coexpressed genes, or searching for combinations of known transcription factor binding sites (TFBSs) (reviewed in [12]). Even these sophisticated sequence analytical approaches can still be quite error prone as shown by Halfon *et al.* [32].

Taken together, these comparative observations point out 2 major advantages of the *C. intestinalis* system for *cis*-regulatory study. First, non-coding sequence conservation comparisons with *C. savignyi,* are at a fortuitous genetic distance to have a high probability of locating functional CREs. These *C. intestinalis-C. savignyi* comparisons consistently identify CNEs that correspond with CREs. Mammals and other chordates, such as amphioxus, do not have such phylogenetically well-positioned sequenced comparison taxa for this purpose. Second, the close proximity of *C. intestinalis *CREs to the transcription start site means that only a few kb of DNA must be scanned experimentally to have a high probability of recovering the major CREs for any gene. This is certainly not the case for the mouse, and to some extent even for the sea urchin.

### Conserved Non-Coding Sequences That Do Not Show cis--regulatory Activity

Even though phylogenetic or binding site sequence analysis can point the way to locating CREs, many, if not most, CNEs do not correspond with CREs identified in empirical experiments, such as reporter gene assays, or ChIP-Seq. In a study of human conserved sequence elements tested in mouse [33], only 29% of DNA sequences conserved between human and Fugu had positive enhancer activity when placed upstream of a heat shock promoter and tested at stage e11.5. Another approach in the same study scanned for possible forebrain specific enhancer sequences. Only 17% of the new elements found had reporter gene forebrain expression. In the small sample of *C. intestinalis* genes shown here (Figs. **3-5**) the correspondance is much better, 67%, although there are many distal CNEs that were not tested for *cis-*regulatory activity. In addition, two *Ci-FoxAa *CREs did not align with a CNE, and would have been missed if only testing conserved sequence elements.

There are, of course, a number of reasons that a conserved non-coding sequence might be functional, and therefore constrained by purifying selection, but not show CRE activity in a reporter assay [12]. One set of possibilities is that the CNE is actually involved in *cis*-regulation, but does not have activation activity that would drive reporter gene expression. These elements could be repressors, insulators or modifiers that have negative regulatory function, and can only be detected by their effect on another *cis-*regulatory element. A CRE may only have activity in concert with another element, so the individual element shows no activity on its own eg. [34]. The CNE could also be a positive acting CRE, but just operate at a developmental stage not tested. Another possibility is "shadow enhancers" - remote activating elements redundant with other CREs but further from the gene that they act upon [35, 36]. Characterization of these cryptic CREs will depend on more sensitive reporter assays, or more exhaustive assays such as ChIP-Seq, that reveal protein-DNA binding without regard to effects on a promoter.

Apart from these genuine but undetected CREs, are other non-coding functional elements, such as matrix attachment regions, or transcribed non-coding RNAs, such as micro RNAs, or long non-coding RNAs. Identification of the function of these CNEs will rely on completely different types of experimental and bioinformatic approaches than those used for CRE discovery.

### Not All cis-regulatory Activity is Confined to Conserved Sequences

As mentioned above, some CREs found through reporter gene assays do not correspond with CNEs (at least at the same level of sensitivity and genetic distance that reveals other CREs). In addition, in cases where CRE dissections have been done in some detail, even if major CRE activity is found in conserved sequences there are sequences outside those conserved that contribute to the expression pattern. For example, in a detailed study of the *Troponin I* gene in *C. intestinalis* [27], 4 conserved sequences were located within a 400 bp region 5' of the TSS, all of which were required to recapitulate the endogenous expression pattern. However, for maximal expression, intervening less conserved sequence was also required. In another case, *Ci-DllB*, a CNE was identified that accounted for the majority of the endogenous expression pattern ("B1" in Fig. **5A**), but an adjacent non-conserved sequence was capable of acting with a portion of the conserved region to activate a similar expression pattern [37].

## THE *CIONA* MODEL AS A MEANS TO STUDY DETAILS OF *CIS-*REGULATORY ARCHITECTURE AND FUNCTION

Apart from the dissection of the *cis-*regulatory architecture of individual genes, *C. intestinalis *lends itself to both broader and deeper investigations of *cis-*regulation. Two studies may serve to demonstrate the particular advantages of reporter gene assays in this animal. Brown and colleagues [38] produced hundreds of reporter gene constructs and produced thousands of transgenic embryos to dissect the *cis-*regulation of co-expressed muscle-specific genes in *C. intestinalis.* They found that though similar DNA-binding proteins cluster in muscle gene CREs, their arrangement and relative contributions to activation of expression vary greatly. The authors also compared the *cis-*regulation of six orthologous muscle-specific genes in *C. intestinalis vs. C. savignyi, *and found large variations between the relative activity conferred by individual binding sites. The simplicity and rapidity of transient *in vivo* transgenic reporter experiments in *Ciona *contributed to the feasibility of performing the large number of assays required for this study. Other workers have also used the *C. intestinalis *system for study of groups of co-expressed genes *e.g.* [39, 40].

Another study by Lemaire and colleagues built on a previous finding of paired ETS and GATA binding sites in an *Otx *enhancer that confers early neural expression [41]. They then used *in silico *analysis to find 55 occurances of this motif in the *C. intestinalis *genome, and then tested each of these in *in vivo* reporter assays. 19 of the sequences showed enhancer activity, and examination of these CREs revealed a nucleosome exclusion motif that was found to be conserved between *Ciona *and *Drosophila* [42]*.* Once again, the ability to test a large number of reporter transgenes rapidly, along with the compactness of the *C. intestinalis *genome and CREs, enhanced the practicality of this study.

## CONCLUSION

*Cis-*regulatory elements in *C. intestinalis* tend to be located relatively close to the transcription start sites of genes, which may be associated with the small size of its genome. This fact combined with the ease and economy of producing transient transgenic embryos in large numbers, have made it an attractive model system for the study of *cis-*regulation. Comparison of experimentally verified CREs with those found in other animals, such as the mouse, suggests that *C. intestinalis* transcriptional regulation relies on a similar complexity of CREs given its anatomical simplicity. Recent work touched on here makes increasingly sophisticated use of the extensive bioinformatic resources for *Ciona*, coupled with analysis of large numbers of *in vivo *reporter gene experiments, to study fundamental aspects of transcriptional regulation. Future work promises to further exploit the *C. intestinalis* model to advance understanding of the function and evolution of *cis-*regulation.

## Figures and Tables

**Table 1.** Bioinformatic Resources for Ascidian Research

Database	Content	URL	Ref.
Ghost	EST sequences, ISH data, genome browser (*C. intestinalis, *v.1), EST clones	ghost.zool.kyoto-u.ac.jp/SearchGenomekh.html#CDNA	[53, 54]
JGI *C. intestinalis* genome	*C. intestinalis* v. 2 genome browser	genome.jgi-psf.org/Cioin2/Cioin2.home.html	[4]
Whitehead *C. savignyi* genome	*C. savignyi* release 1 genome browser	www.broadinstitute.org/annotation/ciona/index.html	n/a
ANISEED	ESTs, gene models, ISH data, genome browser (*C. intestinalis, *v.1), CRE database	www.aniseed.cnrs.fr/	[55]
Tunicate Portal	Links to tunicate bioinformatic resources	www.tunicate-portal.org	n/a
CiAID	ISH data on adult stages, anatomical resources	ioinfo.s.chiba-u.jp/ciaid/atlas2/jv2.htm	[56]
DBTGR	CRE database	dbtgr.hgc.jp	[57]
CiPRO	Proteomics database	cipro.ibio.jp	[58]
FABA	Interactive anatomical body atlas - embryonic	chordate.bpni.bio.keio.ac.jp/faba/top.html	[59]
FABA2	Interactive anatomical body atlas - after hatching	chordate.bpni.bio.keio.ac.jp/faba2/	[59]
CiTRES	GFP reporter transgenic lines	http://marinebio.nbrp.jp/ciona/index.jsp	[60]

**Table 2.** List of Experimentally Verified Transcription Factor and Signaling Gene Enhancers in *Ciona*

Ciona Gene	CRE	Expression Pattern	Ref.
*Ci-achaete-scute-a-like2*	A	Anterior ant. sens. ves., mesenchyme, tail muscles	[55]+
*Ci-ADMP*	A	CNS, epidermis, mesoderm	[55]+
*Ci-AP2-like2*	A	Tail epidermis	[55]+
*Ci-AP4*	A	Mesenchyme, muscle	[55]
	B	Mesenchyme, muscle	
*Ci-Brachyury*	A	Notochord	[8]
*Ci-chordin*	A	CNS, mesoderm	[55]
*Ci-COE*	A	Mesenchyme, muscle	[42]
*Ci-Delta2*	A	Epidermis, siphon primordia, TLCs, TVCs	[55]
*Ci-derriere-like*	A	Epidermis	[55]+
*Ci-DllA*	A1	Anterior neuroectoderm at tailbud stage	[61] Irvine lab unpubl.
*Ci-DllB*	B1	Pan-animal hemisphere at gastrula stage	[37]
*Ci-DMRT1*	A	CNS, mesenchyme, tail muscles, palps	[55]+
*Ci-ELK1*	A	CNS, notochord, muscles, endoderm	[55]+
	B	Mesenchyme, b-epidermis, nerve cord and b-muscle	
	C	a6.5 & b6.5 at 110-cell stage	
*Ci-Emx*	A	Silencer	[55]+
*Ci-EphrinA-c*	A	Head endoderm, notochord	[55]+
*Ci-EphrinA-d*	A	Ectoderm and mesenchyme	[55]+
*Ci-ERF-a*	A	a6.5 & b6.5	[55]+
*Ci-ets*	A	Tail muscles, mesenchyme, part of brain	[55]+
*Ci-Ets97D*	A	Mesenchyme, notochord	[55]+
*Ci-Eya*	A	Epidermis, mesench., neurohyp., palps	[40]
*Ci-fog*	A	Pan-animal hemisphere at 32-cell stage	[62]
*Ci-FoxAa*	A	Lateral CNS	[48]
	B	CNS	
	C	Autoregulation	
	D	Notochord & endoderm	
	E	Ectopic epidermis	
*Ci-FoxB*	A	Notochord, epidermis	[55]+
	B	Mesenchyme, neck, muscle, visc. ganglion	
*Ci-FoxC*	A	Palps, sensory vesicle	[55]+
*Ci-FoxD*	A	A5.1, A5.2, B5.1 lineages	[63]
*Ci-FoxF*	A	Trunk ventral cells at tailbud stage	[64]
*Ci-FoxN2/3*	A	Mesenchyme, tail muscles	[55]+
	B	CNS, mesenchyme, epidermis	
*Ci-orphanFox1*	A	Mesenchyme	[55]+
*Ci-GATAb*	A	a-line ectoderm	[55]+
	B	Mesenchyme	
*Ci-Hes-a*	A	Head endoderm, endodermal strand, epidermis	[55]+
*Ci-Hndx*	A	Endoderm, trunk lateral & trunk ventral cells	[65]
*Ci-Hox1*	A	Epidermis, neural tube	[66]
	B	RA response element	
*Ci-Hox3*	A	Brain	[67]
*Ci-Irx-B*	A	Endoderm, palps, tail epidermis	[55]+
*Ci-KLF1/2/4*	A	Endodermal strand, mesenchyme, tail muscles	[55]+
*Ci-Lhx3*	A	Mesenchyme (B8.5 & B7.7 lines), muscle	[55]+
*Ci-meis*	A	Sensory vesicle, tail muscle	[57]
*Ci-mesp*	A	B7.5 cells at 112-cell stage	[68]
*Ci-Msxb*	A	CNS	[69]
	B	Pharynx	
	C	Ventral epidermis	
*Ci-neurogenin*	A	Nerve cord, muscle, mesenchyme	[55]+
*Ci-Nodal*	A	Notochord, tail epidermis, ventral head epidermis	[55]+
	B	b8.17, b8.18, b8.19, b8.20 at 112-cell stage	
*Ci-NPP*	A	Head endoderm. TVCs	[65]
*Ci-Otx*	A	Neuroectoderm from 32-cell stage (“a-element”)	[41]
*Ci-paraxis*	A	Primary muscle lineage	[70]
*Ci-Pax6*	UB	Nerve cord & sensory vesicle amplifier	[44]
	UA	Sensory vesicle	
	I1	Photoreceptors & nerve cord amplifier	
	I4	Ectopic repression	
*Ci-Pitx*	A	Neurohypophysis	[71]
	B	Epidermis	
*Ci-RAR*	A	Muscle	[72]
	B	CNS	
	C	Epidermis (“E element”)	
*Ci-Rora*	A	a-line ectoderm at gastrula stage (“AS2”	[73]
*Ci-Rorb*	A	a-line ectoderm at gastrula stage (“BS1”)	[73]
	B	a-line neural ectoderm gastrula stage (“BS4”)	
	C	Neural gland of adult & early repressors (“BL”)	
*Ci-Rx*	A	CNS	[74]
*Ci-sFRP1/5*	A	Anterior gastrula ectoderm	[75]
*Ci-sna*	A	B4.1 lineage	[76]
*Ci-SoxB1*	A	Ectoderm	[55]+
	B	Mesenchyme	
*Ci-SoxC*	A	Mesenchyme, muscle, epidermis (only midline), epidermal neruons, palps and a part of brain	[55]+
*Ci-Tbx6b*	A	Muscle??	[70]
*Ci-TTF1 (Nkx2-1)*	A	Endoderm	[77]
*Ci-Trim2/3*	A	Anterior neural precursors (weak)	[55]+
*Ci-Unc4A*	A	Notochord	[55]+
*Ci-Wnt5*	A	Muscle precursors	[55]+
*Ci-ZicL-B*	A	A6.2 & A6.4 lineages	[78]
	B	B6.2 & B6.4 lineages & later A-line notochord, nerve cord & muscle	
*Ci-Znf(C2H2)-24*	A	Mesenchyme, endodermal strand, muscle	[55]+
*Ci-Znf(C3H)*	A	CNS, mesenchyme, notochord, tail muscles, palps	[55]+

* This element not shown on alignment.

+ Data from Y. Ohtsuka, unpublished, accessed on www.aniseed.cnrs.fr.
