# Supplementary material for: Study of Cis-regulatory Elements in the Ascidian Ciona intestinalis
Source: Curr Genomics. 2013 Mar;14(1):56–67. doi: 10.2174/138920213804999192 (PMC3580780; doi:10.2174/138920213804999192)
Supplement: Supplementary file 1 [file CG-14-56_SD1.pdf]

**Table S1. Experimentally Verified *cis*-regulatory Elements in *Ciona intestinalis*. Data Derived from Comparison of the *C. intestinalis* Genome v.1 or v.2 As Noted [1] and Gene Models and *cis*regulatory Information from the ANISEED Database [2] and Other References As Noted**

| Ciona gene | Gene model | Genome | Trx. start site | GRE | Locus | Mean distance to TSS | Expression pattern | Reference |
|---|---|---|---|---|---|---|---|---|
| *Ci-achaete-scute-a-like2* | ci0100148570 | v.1 | scaffold_667: 25699 | A | 24704-25641 | -0.53 | anterior ant. sens. ves., mesenchyme, tail muscles | Y. Ohtsuka, ANISEED |
| *Ci-ADMP* | KH.C2.421.v1.A.nonSL4-1 | v.2 | scaffold_213: 107269 | A | 106593-107007 | -0.47 | CNS, epidermis, mesoderm | ANISEED |
| *Ci-AP2-like2* | KH.C7.43.v1.A.nonSL1-1 | v.1 | scaffold_107: 140192 | A | 141387-141664 | -1.33 | tail epidermis | ANISEED |
| *Ci-AP4* | KH.C14.448.v1.A.ND1-1 | v.1 | scaffold_73: 110517 | A | 109928-110292 | +0.41 | mesenchyme, muscle | Y. Ohtsuka, ANISEED |
| | | | | B | 110613-110767 | -0.17 | mesenchyme, muscle | |
| *Ci-Brachyury* | ci0100134430 | v.2 | scaffold_162: 25953 | A | 25588-25953 | -0.21 | notochord | [3] |
| *Ci-chordin* | KH.C6.145.v1.A.ND1-1 | v.1 | scaffold_8: 356794 | A | 356873-357169 | -9.6 | CNS, mesoderm | ANISEED |
| *Ci-COE* | ci0100133553 | v.1 | scaffold_229: 99269 | A | 108589-109151 | -0.23 | mesenchyme, muscle | [4] |
| *Ci-Delta2* | KH.L50.6.v1.A.SL1-1 | v.1 | scaffold_170: 33066 | A | 32545-33133 | -0.23 | epidermis, siphon primordia, TLCs, TVCs | Y. Ohtsuka, ANISEED |
| *Ci-derriere-like* | KH.C4.547.v1.A.nonSL2-1 | v.1 | scaffold_123: 191879 | A | 191627-191803 | -016 | epidermis | [4] |
| *Ci-DllA* | KH.C7.346.v1.A.SL1-1 | v.2 | chr_07q: 1163893 | A | 1162089-1163705 | -0.41 | anterior neuroectoderm at tailbud stage | [5] |
| *Ci-DllB* | KH.C7.243.v1.A.ND1-1 | v.2 | chr_07q: 1170654 | A | 1170826-1171284 | -0.40 | pan-animal hemisphere at gastrula stage | [6] |
| *Ci-DMRT1* | KH.S544.3.v1.A.nonSL2-1 | v.2 | chr_05q: 5020647 use v1 for gene map | A | 5020616-5021001 | -0.16 | CNS, mesenchyme, tail muscles, palps | [4] |
| *Ci-ELK1* | KH.C8.247.v2.A.SL2-1 | v.1 | scaffold_3: 773886 | A | 773052-773898 | -0.41 | CNS, notochord, muscles, endoderm | [4] |
| | | | | B | 777331-777667 | +3.61 | mesenchyme, b-epidermis, nerve cord and b-muscle | |
| | | | | C | 781042-781175 | +7.22 | a6.5 & b6.5 at 110-cell stage | |
| *Ci-Emx* | ci0100151065 | v.1 | scaffold_402: 9128 | A | 9240-9653 | -0.32 | silencer | [4] |
| *Ci-EphrinA-c* | KH.C3.52.v1.A.SL2-1 | v.1 | scaffold_208: 134793 | A | 134817-135556 | -0.39 | head endoderm, notochord | [4] |
| *Ci-EphrinA-d* | KH.C3.716.v1.A.SL1-1 | v.1 | scaffold_208: 140900 | A | 140976-141642 | -0.41 | ectoderm and mesenchyme | [4] |
| *Ci-ERF-a* | KH.C4.366.v1.A.ND1-1 | v.1 | scaffold_6: 551867 | A | 562958-563160 | -11.19 | a6.5 & b6.5 | [4] |

| Ciona gene | Gene model | Genome | Trx. start site | GRE | Locus | Mean distance to TSS | Expression pattern | Reference |
|---|---|---|---|---|---|---|---|---|
| *Ci-ets* | KH.C10.113.v1.A.SL1-1 | v.1 | scaffold_344: 72641 | A | 71681-72434 | -0.58 | tail muscles, mesenchyme, part of brain | [4] |
| *Ci-Ets97D* | ci0100130209 | v.1 | scaffold_28: 55481 | A | 53642-54159 | -1.58 | mesenchyme, notochord | [4] |
| *Ci-Eya* | ci0100137801 | v.1 | scaffold_368: 2214* | A | 4544-5782 | +2.95 | epidermis, mesench., neurohyp., palps | [7] |
| *Ci-fog* | ci0100149797 | v.1 | scaffold 9: 331369 | A | 331487-331828 | -0.29 | pan-animal hemisphere at 32-cell stage | [8] |
| *Ci-FoxAa* | KYOTOGRAIL2005.63.50.1 | v.1 | scaffold_63: 269243 | A | 269751-269811 | -0.54 | lateral CNS | [9] & [2] |
| | | | | B | 270101-270571 | -1.09 | CNS | |
| | | | | C | 270572-270830 | -1.49 | autoregulation | |
| | | | | D | 270831-270971 | -1.66 | notochord & endoderm | |
| | | | | E | 270971-271801 | -2.14 | ectopic epidermis | |
| *Ci-FoxB* | ci0100145669 | v.2 (v.1) | chr_04q: 4634207 (sc_129: 70240) | A | 4630261-4630689 (73762-74192) | -3.73 | notochord, epidermis | [4] |
| | | | | B | 4633083-4633346 (71106-71369) | -0.99 | mesenchyme, neck, muscle, visc. ganglion | |
| *Ci-FoxC* | KH.L57.25.v1.A.SL1-1 | v.1 | scaffold_333: 73634 | A | 73706-74127 | -0.28 | palps, sensory vesicle | [4] |
| *Ci-FoxD* | ci0100146995 | v.2 | chr_08q: 2997576 | A | 2996334-2996492 | -1.16 | A5.1, A5.2, B5.1 lineages | [10] |
| *Ci-FoxF* | KH.C3.170.v1.A.SL1-1 | v.1 | scaffold_56: 139736 | A | 138750-138963 | -0.88 | trunk ventral cells at tailbud stage | [11] |
| *Ci-FoxN2/3* | KH.L13.24.v1.A.SL1-1 | v.1 | scaffold_160: 140569 | A | 138298-138668 | -2.09 | mesenchyme, tail muscles | [4] |
| | | | | B | 139364-140470 | -0.65 | CNS, mesenchyme, epidermis | |
| *Ci-orphanFox1* | KH.C14.520.v1.A.ND1-1 | v.1 | scaffold_73: 311622 | A | 314878-315158 | -3.4 | mesenchyme | [4] |
| *Ci-GATAb* | KH.S696.1.v1.A.ND1-1 | v.2 | scaffold_95: 471757 | A | 469845-470050 | -1.81 | a-line ectoderm | [4] |
| | | | | B | 470580-470781 | -1.08 | mesenchyme | |
| *Ci-Hes-a* | KH.L34.9.v1.A.ND2-1 | v.1 | scaffold_275: 72799 | A | 74194-74407 | -1.5 | head endoderm, endodermal strand, epidermis | [4] |
| *Ci-Hndx* | ci0100140298 | v.2 | chr_01q: 4071779 | A | 4071838-4074717 | -1.5 | endoderm, trunk lateral & trunk ventral cells | [12] |

| Ciona gene | Gene model | Genome | Trx. start site | GRE | Locus | Mean distance to TSS | Expression pattern | Reference |
|---|---|---|---|---|---|---|---|---|
| *Ci-Hox1* | KH.L171.16.v1.A.SL2-1 | v.1 | scaffold_148: 187453 | A | 185772-183869* | +2.63 | epidermis, neural tube | [13] |
| | | | | B | 187959-187795 | -0.42 | RA response element | |
| *Ci-Hox3* | KH.C1.38.v2.A.SL2-1 | v.1 | scaffold_87: 133201 | A | 133815-133895 | -1.91 | brain | [14] |
| *Ci-Irx-B* | KH.C11.485.v1.A.SL1-1 | v.1 | scaffold_12: 254812 | A | 256394-257214 | -1.99 | endoderm, palps, tail epidermis | [4] |
| *Ci-KLF1/2/4* | KH.C5.154.v1.A.ND1-1 | v.2 | scaffold_86: 294192 | A | 294494-295143 | -0.63 | endodermal strand, mesenchyme, tail muscles | [4] |
| *Ci-Lhx3* | ci0100130659 | v.2 | chr_13q: 2001122 | A | 2005216-2005510 | -4.24 | mesenchyme (B8.5 & B7.7 lines), muscle | [4] |
| *Ci-meis* | KH.C10.174.v1.A.SL2-1 | v.1 | scaffold_38: 68952 | A | 62492-66776 | -4.32 | sensory vesicle, tail muscle | [15] |
| *Ci-mesp* | ci0100138454 | v.2 | chr_03q: 2495630 | A | 2495593-2495781 | -0.06 | B7.5 cells at 112-cell stage | [16] |
| *Ci-Msxb* | KYOTOGRAIL2005.29.6.1 | v.2 | chr_02q: 6152285 | A | 6152058-6152201 | -0.16 | CNS | [17] |
| | | | | B | 6151828-6152058 | -0.34 | pharynx | |
| | | | | C | 6149739-6151467 | -1.68 | ventral epidermis | |
| *Ci-neurogenin* | KH.C6.129.v1.A.nonSL6-1 | v.1 | scaffold_8: 77488 | A | 77550-77932 | -0.25 | nerve cord, muscle, mesenchyme | [4] |
| *Ci-Nodal* | KH.L106.16.v1.A.SL1-1 | v.1 | scaffold_490: 16680 | A | 18759-18940 | -2.17 | notochord, tail epidermis, ventral head epidermis | [4] |
| | | | | B | 18415-18561 | -1.81 | b8.17, b8.18, b8.19, b8.20 at 112-cell stage | |
| *Ci-NPP* | KH.C2.514.v2.A.ND1-2 | v.1 | scaffold_240: 82362 | A | 83459-82403 | -0.57 | head endoderm. TVCs | [12] |
| *Ci-Otx* | KH.C4.84.v2.A.SL4-1 | v.2 | chr_04a: 4277070 | A | 4278481-4278604 | -1.47 | neuroectoderm from 32-cell stage ("a-element") | [18] |
| *Ci-paraxis* | KH.S781.11.v1.A.SL1-1 | v.1 | scaffold_781: 19180 | A | 18941-19081 | -0.17 | primary muscle lineage | [19] |
| *Ci-Pax6* | KH.C9.68.v1.A.ND1-1 | v.2 | chr_09q: 3323726 | A | 3321375-3321811 | -2.13 | nerve cord & sensory vesicle amplifier | [20] |
| | | | | B | 3321812-3322012 | -1.81 | sensory vesicle | |
| | | | | C | 3324100-3325800 | +1.22 | photoreceptors & nerve cord amplifier | |
| | | | | D | 3327800-3329900 | +5.12 | ectopic repression | |
| *Ci-Pitx* | KH.L153.79.v2.A.SL2-1 | v.1 | scaffold_121: 181422 | A | 170509-170703 | -10.82 | neurohypophysis | [21] |

| Ciona gene | Gene model | Genome | Trx. start site | GRE | Locus | Mean distance to TSS | Expression pattern | Reference |
|---|---|---|---|---|---|---|---|---|
| | | | | B | 183532-183691* | +2.19 | epidermis | |
| *Ci-RAR* | KH.C9.580.v2.A.SL2-1 | v.2 | chr_09q: 1244192 | A | 1244386-1244486 | -0.24 | muscle | [22] |
| | | | | B | 1244637-1244979 | -0.62 | CNS | |
| | | | | C | 1244980-1245493 | -1.04 | epidermis ("E element") | |
| *Ci-Rora* | KH.C8.101.v1.A.ND2-1 | v.2 | chr_08q: 3106486 | A | 3107026-3107144 | -0.60 | a-line ectoderm at gastrula stage ("AS2" | [23] |
| *Ci-Rorb* | KH.C8.737.v1.A.SL1-1 | v.2 | chr_08q: 3083185 | A | 3083629-3083788 | -0.52 | a-line ectoderm at gastrula stage ("BS1") | [23] |
| | | | | B | 3082245-3082494 | +0.82 | a-line neural ectoderm gastrula stage ("BS4") | |
| | | | | C* | 3084832-3078865 | -1.34 | neural gland of adult & early repressors ("BL") | |
| *Ci-Rx* | KH.C12.152.v1.A.SL1-1 | v.1 | scaffold_84: 162648 | A | 163276-163080 | -0.53 | CNS | [24] |
| *Ci-sFRP1/5* | KYOTOGRAIL2005.148.4.1 | v.1 | scaffold_148: 32589 | A | 33924-34041 | -1.39 | anterior gastrula ectoderm | [25] |
| *Ci-sna* | KH.C3.751.v1.C.SL1-1 | v.2 | chr_03q: 4125486 | A | 4125656-4126157 | -0.42 | B4.1 lineage | [26] |
| *Ci-SoxB1* | KH.C1.99.v1.A.SL1-1 | v.1 | scaffold_92: 251079 | A | 251295-251584 | -0.36 | ectoderm | [4] |
| | | | | B | 252621-252990 | -1.73 | mesenchyme | |
| *Ci-SoxC* | KH.C7.523.v1.A.SL1-1 | v.2 | chr_07q: 3615183 | A | 3614302-3614949 | -0.56 | mesenchyme, muscle, epidermis (only midline), epidermal neruons, palps and a part of brain | [4] |
| *Ci-Tbx6b* | KH.S654.3.v1.A.nonSL1-1 | v.1 | scaffold_654 26946 | A | 27239-27383 | -0.37 | muscle?? | [19] |
| *Ci-TTF1 (Nkx2-1)* | KH.C10.338.v1.A.nonSL1-1 | v.1 | scaffold_371: 37135 | A | 36583-36807 | -0.44 | endoderm | [27] |
| *Ci-Trim2/3* | KH.C8.863.v1.A.SL1-1 | v.1 | scaffold_1: 627630 **need to update gene model on Vista** | A | 627756-627878 | +0.19 | anterior neural precursors (weak) | [4] |
| *Ci-Unc4A* | KH.C5.34.v1.A.SL1-1 | v.1 | scaffold_50: 113746 | A | 108889-108759 | -4.92 | notochord | [4] |
| *Ci-Wnt5* | KH.L152.45.v1.A.SL1-1 | v.1 | scaffold_248: 119170 | A | 118387-118506 | -0.72 | muscle precursors | [4] |
| *Ci-ZicL-B* | ci0100145774 | v.1 | scaffold_577: 39659 | A | 39700-39997 | -0.19 | A6.2 & A6.4 lineages | [28] |
| | | | | B | 39998- | -0.57 | B6.2 & B6.4 | |

| Ciona gene | Gene model | Genome | Trx. start site | GRE | Locus | Mean distance to TSS | Expression pattern | Reference |
|---|---|---|---|---|---|---|---|---|
| | | | | | 40451 | | lineages & later A-line notochord, nerve cord & muscle | |
| *Ci-Znf(C2H2)-24* | KH.C5.154.v1.A.ND1-1 | v.1 | scaffold_86: 294192 | A | 294494-295143 | -0.63 | mesenchyme, endodermal strand, muscle | [4] |
| *Ci-Znf(C3H)* | KH.C4.182.v1.A.nonSL3-1 | v.2 | chr_14q: 884369 use v1 for gene map | A | 884346-884581 | -0.09 | CNS, mesenchyme, notochord, tail muscles, palps | [4] |

* This element not shown on alignment.

**Table S2. Experimentally Verified *cis*-regulatory Elements in Mouse. Data Were Derived from the References Noted**

| Mouse gene | CRE name | 5' end | 3' end | Mean distance to TSS | Expression pattern | Notes | Reference |
|---|---|---|---|---|---|---|---|
| *Dll1* | msdII | -.8 | -.08 | -0.44 | Somites | | [29] |
| | H2 | -1.16 | -1.0 | -1.08 | Dors. sp. cord | | |
| | msd | -12.76 | -1.16 | -6.96 | Somites | | |
| | H1 | -3.04 | -2.76 | -2.9 | Vent. sp. cord, sp. ganglia | | |
| *Dlx1* | URE1 | -17.6 | -17 | -17.3 | not described | 1 | [30] |
| | URE2 | -12.9 | -12 | -12.45 | Forebrain | | |
| | I12a | +6.4 | +6.95 | +6.68 | Branchial arches | | [31] |
| | I12b | +8.4 | +8.8 | +8.6 | Forebrain | | |
| *Fgf3* | n/a | -12.76 | -7.1 | -9.93 | Brain, ectoderm, ganglia | | [32] |
| *Foxa2* | NE | -14 | -13.5 | -13.75 | Notochord | | [33] |
| | FPE | +9.5 | +10.0 | +9.75 | Floor plate | | [34] |
| *Gli3* | CNE1 | +26 | +26.95 | +26.48 | Brain, sp. cord, face | 2 | [35] |
| *Gata1* | -25/6 | -25.8 | -24.2 | -25.0 | Erythrocytes | | [36] |
| | -3.5 | -3.8 | -3.3 | -3.55 | Erythrocytes | | |
| | +3.5 | +3.2 | +3.8 | +3.5 | Erythrocytes | | |
| *Gata4* | G2 | -38.7 | -37.7 | -38.2 | Lateral mesoderm | 5 | [37] |
| | G9 | -93.77 | -93.36 | -93.57 | Endocardium | | [38] |
| | G8 | -80.95 | -80.73 | -80.84 | Endoderm | | [39] |
| | Prox | -0.18 | -0.07 | -0.13 | Gonads | | [40] |
| *Hb9* | A | -8.0 | -7.87 | -7.94 | Motor neurons | | [41] |
| *Hoxa1* | I | -6.7 | -5.3 | -6.0 | Rhombomere 4 | | [42] |
| | II | -5.3 | -3 | -4.15 | Rhombomere 2, somites | | |
| | III | +4.39 | +4.96 | +4.68 | Notochord, sp. cord | | |
| *Hoxa2* | n.c. | -2.3 | -1.85 | -2.08 | Neural crest | | [43] |
| | r3/5 | -2.65 | -2.3 | -2.48 | Rhombomeres 3 & 5 | | |
| | exon | +1.69 | +1.78 | +1.74 | Rhombomere 2 | | [44] |
| *Hoxa3* | r5/6 | -4.64 | -4.23 | -4.44 | Rhombomeres 5 & 6 | | [45] |

| Mouse gene | CRE name | 5' end | 3' end | Mean distance to TSS | Expression pattern | Notes | Reference |
|---|---|---|---|---|---|---|---|
| *Hoxa7* | AX470 | -2.1 | -1.6 | -1.85 | somites, sp. cord, sp. ganglia | | [46] |
| *Hoxb1* | RAIDR5 | +7.2 | +6.5 | +6.85 | RA response | | [47] |
| *Hoxb3* | Ia | -0.6 | -0.1 | -0.35 | Rhombomere 5 | 4 | [48][49] |
| | Ib | +1.0 | +1.4 | +1.2 | Mesoderm | | |
| | IIIa | +11.9 | +12.4 | +12.15 | Hindbr. & neural crest | | |
| | IVa | +17.4 | +17.9 | +17.65 | Rhombomere 5 | | |
| | IVb | +18.4 | +18.9 | +18.65 | Neural and mesoderm | | |
| | Va | +19.4 | +19.8 | +19.6 | Mesoderm | | |
| *Hoxb4* | e | +0.95 | +1.85 | +1.4 | sp. cord, somites | | [50] |
| | A | +3.4 | +6.4 | +4.9 | sp. cord | | |
| | ENE | +6.2 | +6.45 | +6.33 | Neural | | [51] |
| | LNE | +5.0 | +5.25 | +5.13 | Neural | | |
| *Hoxb6* | early | -2.47 | -2.22 | -2.35 | Lat. plt. mesoderm | | [52] |
| *Hoxc8* | ENT | -3.85 | -3.34 | -3.6 | Sp. cord, mesoderm | | [53] |
| | Late | +11 | +19 | +15 | Sp. cord, somites | | |
| *Hoxc9* | CSE1 | +2.16 | +2.27 | +2.22 | Sp. cord | | [54] |
| | intron | +0.81 | +2.24 | +1.53 | Sp. cord | | |
| *Hoxd4* | Autoreg | -2.0 | -1.71 | -1.85 | Somites | | [55] |
| | A | +3.6 | +4.4 | +4.0 | CNS | | [56] |
| *Msx1* | DE | -4.43 | -4.18 | -4.31 | 1st arch, nasal epithelium | | [57] |
| | PE | -2.39 | -2.31 | -2.35 | Myotome, limb mesench., other | | |
| *Msx2* | n/a | -3.86 | -3.30 | -3.58 | BMP response | | [58] |
| | AER | -0.50 | -0.44 | -0.47 | Apical ectodermal ridge | | [59] |
| *Myf5* | EEE | -6.1 | -5.6 | -5.85 | Epaxial somites | 6 | [60] |
| | PAE | -2.7 | -1.7 | -2.2 | Branchial arches | | |
| | CNSE | -1.7 | -0.1 | -0.9 | CNS | | |
| | n/a | +0.5 | +3.3 | +1.9 | Hypaxial somites, branchial arches | | |
| | Myf5-IV | -57.7 | -55.9 | -56.8 | Myotome, limb muscles, brain | | [61][62] |
| | | -16.8 | -16.2 | -16.5 | Thoracic myotome | | [63] |
| *Myf6* | n/a | -6.5 | -5.6 | -6.05 | Epaxial myotome | 6 | [64] |
| | A17 | -8.2 | -7.6 | -7.9 | Thoracic myotome | | [65] |
| *Nkx2-5* | n/a | -10.7 | -8.5 | -9.6 | Rt. ventricle | | [66] |
| | n/a | -5.0 | -4.0 | -4.5 | Rt. ventricle | | |
| | n/a | -3.5 | -1.5 | -2.5 | Thyroid, spleen, stomach | | [67] |
| *Nodal* | NDE | -9.5 | -8.7 | -9.1 | Node | | [68] |
| | ASE | +1.0 | +1.34 | +1.17 | Assym. in lat. plt. meso. | | |
| | LSE | -4.5 | -4.0 | -4.25 | Left side lat. plt. meso. | | |
| *Oct4* | PE | -1.13 | -.89 | -1.01 | ES cells | 3 | [69] |
| | DE | -2.1 | -1.29 | -1.70 | ES cells | | |

| Mouse gene | CRE name | 5' end | 3' end | Mean distance to TSS | Expression pattern | Notes | Reference |
|---|---|---|---|---|---|---|---|
| *Otx2* | CNE1 | -3.1 | -2.76 | -2.93 | Visc. endo., ceph. mesench. | | [70][71] |
| | VE cis | -1.8 | 0 | -0.9 | Visc. endo. | | |
| | 2 | -5.06 | -4.91 | -4.99 | Retina | | |
| | FM | -71.05 | -70.7 | -70.88 | Forebrain & midbrain | | |
| | FM2 | +125.6 | +126.6 | +126.1 | Forebrain & midbrain | | |
| | AN | -91.2 | -88.9 | -90.05 | Anterior neuroectoderm | | |
| *Pax6* | A | -4.24 | -4.0 | -4.12 | Pancreas | | [72][73] |
| | B | -3.81 | -3.64 | -3.73 | Lens | | |
| | D/α | +13.27 | +13.72 | +13.50 | Retina, spinal cord | | |
| | P0 | -2.35 | -1.9 | -2.13 | Retina, pancreas | | [74] |
| | P1 | +3.45 | +3.65 | +3.55 | Photoreceptors | | |
| | 7CE1 | +17.7 | +18.3 | +18.0 | Lens | | [75] |
| | 7CE2 | +19.2 | +19.77 | +19.49 | Brain, heart | | |
| *Six1* | 1-14 | -1.91 | -1.35 | -1.63 | Pre-placodal region | | [76] |
| *Sox2* | | -4.1 | -3.3 | -3.7 | ES cells | | [77] |
| | | -2.4 | -1.9 | -2.15 | ES cells | | |
| | | +3.6 | +4.4 | +4.0 | ES cells | | |
| *Sox10* | MCS2 | -1.90 | -1.21 | -1.56 | Neurons, craniofac., otic ves. | | [78] |
| | MCS3 | -10.29 | -9.53 | -9.91 | Schwann cells | | |
| | MCS4 | -29.32 | -28.51 | -28.92 | Neurons, craniofac., melanocytes | | |

Notes:

1 - I12a & I12b enhancers located from Dlx1 TSS.

2 - Human elements tested in mouse (conserved in mouse sequence).

3 - a.k.a. *Oct3, Pou5f1*.

4 - Coordinates based on a 4-exon gene model.

5 - Coordinates based on NCBI37/mm9 assembly UCSC gene model.

6 - Some enhancers shared between *Myf5* and *Myf6* (a.k.a *MRF4*), e.g. *Myf5* -16.8 and *Myf6* -8.2 elements.

## SUPPLEMENTAL REFERENCES


[1] Dehal, P.; Satou, Y.; Campbell, R. K.; Chapman, J.; Degnan, B.; De Tomaso, A.; Davidson, B.; Di Gregorio, A.; Gelpke, M.; Goodstein, D. M.; Harafuji, N.; Hastings, K. E. M.; Ho, I.; Hotta, K.; Huang, W.; Kawashima, T.; Lemaire, P.; Martinez, D.; Meinertzhagen, I. A.; Necula, S.; Nonaka, M.; Putnam, N.; Rash, S.; Saiga, H.; Satake, M.; Terry, A.; Yamada, L.; Wang, H. G.; Awazu, S.; Azumi, K.; Boore, J.; Branno, M.; Chin-bow, S.; DeSantis, R.; Doyle, S.; Francino, P.; Keys, D. N.; Haga, S.; Hayashi, H.; Hino, K.; Imai, K. S.; Inaba, K.; Kano, S.; Kobayashi, K.; Kobayashi, M.; Lee, B. I.; Makabe, K. W.; Manohar, C.; Matassi, G.; Medina, M.; Mochizuki, Y.; Mount, S.; Morishita, T.; Miura, S.; Nakayama, A.; Nishizaka, S.; Nomoto, H.; Ohta, F.; Oishi, K.; Rigoutsos, I.; Sano, M.; Sasaki, A.; Sasakura, Y.; Shoguchi, E.; Shin-i, T.; Spagnuolo, A.; Stainier, D.; Suzuki, M. M.; Tassy, O.; Takatori, N.; Tokuoka, M.; Yagi, K.; Yoshizaki, F.; Wada, S.; Zhang, C.; Hyatt, P. D.; Larimer, F.; Detter, C.; Doggett, N.; Glavina, T.; Hawkins, T.; Richardson, P.; Lucas, S.; Kohara, Y.; Levine, M.; Satoh, N.; Rokhsar, D. S., The draft genome of Ciona intestinalis: Insights into chordate and vertebrate origins. *Science,* **2002**, 298, (5601), 2157-2167.

[2] Tassy, O.; Dauga, D.; Daian, F.; Sobral, D.; Robin, F.; Khoueiry, P.; Salgado, D.; Fox, V.; Caillol, D.; Schiappa, R.; Laporte, B.; Rios, A.; Luxardi, G.; Kusakabe, T.; Joly, J. S.; Darras, S.; Christiaen, L.; Contensin, M.; Auger, H.; Lamy, C.; Hudson, C.; Rothbacher, U.; Gilchrist, M. J.; Makabe, K. W.; Hotta, K.; Fujiwara, S.; Satoh, N.; Satou, Y.; Lemaire, P., The ANISEED database: Digital representation, formalization, and elucidation of a chordate developmental program. *Genome Res.,* **2010,** 20, (10), 1459-1468.

[3] Corbo, J. C.; Levine, M.; Zeller, R. W., Characterization of a notochord-specific enhancer from the *Brachyury* promoter region of the ascidian, *Ciona intestinalis*. *Development,* **1997,** 124, 589-602.

[4] Khoueiry, P.; Rothbacher, U.; Ohtsuka, Y.; Daian, F.; Frangulian, E.; Roure, A.; Dubchak, I.; Lemaire, P., A cis-Regulatory Signature in Ascidians and Flies, Independent of Transcription Factor Binding Sites. *Curr. Biol.,* **2010,** 20, (9), 792-802.

[5] Harafuji, N.; Keys, D. N.; Levine, M., Genome-wide identification of tissue-specific enhancers in the *Ciona* tadpole. *Proc. Natl. Acad. Sci. USA,* **2002,** 99, 6802-6805.

[6] Irvine, S. Q.; Vierra, D. A.; Millette, B. J.; Blanchette, M. D.; Holbert, R. E., Expression of the *Distalless-B* gene in *Ciona* is regulated by a pan-ectodermal enhancer module. *Dev. Biol.,* **2011,** 353, 432-439.

[7] Haeussler, M.; Jaszczyszyn, Y.; Christiaen, L.; Joly, J. S., A cis-Regulatory Signature for Chordate Anterior Neuroectodermal Genes. *PLoS Genet.,* **2010,** 6, (4).

[8] Rothbacher, U.; Bertrand, V.; Lamy, C.; Lemaire, P., A combinatorial code of maternal GATA, Ets and beta-catenin-TCF transcription factors specifies and patterns the early ascidian ectoderm. *Development,* **2007,** 134, (22), 4023-4032.

[9] Di Gregorio, A.; Corbo, J.; Levine, M., The regulation of forkhead/HNf-3 beta expression in the Ciona embryo. *Dev. Biol.,* **2001,** 229, 31-43.

[10] Mita, K.; Fujiwara, S., Nodal regulates neural tube formation in the Ciona intestinalis embryo. *Dev. Genes Evol.,* **2007,** 217, (8), 593-601.
[11] Beh, J.; Shi, W.; Levine, M.; Davidson, B.; Christiaen, L., FoxF is essential for FGFinduced migration of heart progenitor cells in the ascidian Ciona intestinalis. *Development,* **2007**, 134, (18), 3297-3305.
[12] Davidson, B.; Levine, M., Evolutionary origins of the vertebrate heart: Specification of the cardiac lineage in Ciona intestinalis. *Proc. Natl. Acad. Sci. USA,* **2003,** 100, (20), 11469-11473.
[13] Kanda, M.; Wada, H.; Fujiwara, S., Epidermal expression of Hox1 is directly activated by retinoic acid in the Ciona intestinalis embryo. *Dev. Biol.,* **2009,** 335, (2), 454-463.
[14] Locascio, A.; Aniello, F.; Amoroso, A.; Manzanares, M.; Krumlauf, R.; Branno, M., Patterning the ascidian nervous system: structure, expression and transgenic analysis of the *CiHox3* gene. *Development,* **1999,** 126, 4737-4748.
[15] Sierro, N.; Kusakabe, T.; Park, K. J.; Yamashita, R.; Kinoshita, K.; Nakai, K., DBTGR: a database of tunicate promoters and their regulatory elements. *Nucleic Acids Res.,* **2006,** 34, D552-D555.
[16] Christiaen, L.; Stolfi, A.; Davidson, B.; Levine, M., Spatio-temporal intersection of Lhx3 and Tbx6 defines the cardiac field through synergistic activation of Mesp. *Dev. Biol.,* **2009,** 328, (2), 552-560.
[17] Russo, M.; Donizetti, A.; Locascio, A.; D'Aniello, S.; Amoroso, A.; Aniello, F.; Fucci, L.; Branno, M., Regulatory elements controlling Ci-msxb tissue-specific expression during Ciona intestinalis embryonic development. *Dev. Biol.,* **2004,** 267, 517-528.
[18] Bertrand, V.; Hudson, C.; Caillol, D.; Popovici, C.; Lemaire, P., Neural tissue in ascidian embryos is induced by FGF9/16/20, acting via a combination of maternal GATA and Ets transcription factors. *Cell,* **2003,** 115, (5), 615-627.
[19] Erives, A., Non-Homologous Structured CRMs from the Ciona Genome. *J. Comput. Biol.,* **2009,** 16, (2), 369-377.
[20] Irvine, S. Q.; Fonseca, V. C.; Zompa, M. A.; Antony, R., Cis-regulatory organization of the Pax6 gene in the ascidian Ciona intestinalis. *Dev. Biol.,* **2008,** 317, (2), 649-659.
[21] Christiaen, L.; Bourrat, F.; Joly, J. S., A modular cis-regulatory system controls isoformspecific pitx expression in ascidian stomodaeum. *Dev. Biol.,* **2005,** 277, (2), 557-566.
[22] Fujiwara, S., Promoter activity of the retinoic acid receptor gene in the Ciona intestinalis embryo. *Dev. Dyn.,* **2005,** 232, (4), 1124-1130.
[23] Auger, H.; Lamy, C.; Haeussler, M.; Khoueiry, P.; Lemaire, P.; Joly, J. S., Similar regulatory logic in Ciona intestinalis for two Wnt pathway modulators, ROR and SFRP-1/5. *Dev. Biol.,* **2009,** 329, (2), 364-373.
[24] D'Aniello, E.; Pezzotti, M. R.; Locascio, A.; Branno, M., Onecut is a direct neuralspecific transcriptional activator of Rx in Ciona intestinalis. *Dev. Biol.,* **2011,** 355, (2), 358-371.
[25] Lamy, C.; Rothbacher, U.; Caillol, D.; Lemaire, P., Ci-FoxA-a is the earliest zygotic determinant of the ascidian anterior ectoderm and directly activates Ci-sFRP1/5. *Development,* **2006,** 133, (15), 2835-2844.
[26] Erives, A.; Corbo, J. C.; Levine, M., Lineage-specific regulation of the Ciona snail gene in the embryonic mesoderm and neuroectoderm. *Dev. Biol.,* **1998,** 194, (2), 213-225.
[27] Fanelli, A.; Lania, G.; Spagnuolo, A.; Di Lauro, R., Interplay of negative and positive signals controls endoderm-specific expression of the ascidian Cititf1 gene promoter. *Dev. Biol.,* **2003,** 263, 12-23.
[28] Anno, C.; Satou, A.; Fujiwara, S., Transcriptional regulation of ZicL in the Ciona intestinalis embryo. *Dev. Genes Evol.,* **2006,** 216, (10), 597-605.
[29] Beckers, J.; Caron, A.; de Angelis, M. H.; Hans, S.; Campos-Ortega, J. A.; Gossler, A., Distinct regulatory elements direct Delta 1 expression in the nervous system and paraxial mesoderm of transgenic mice. *Mech. Dev.,* **2000,** 95, (1-2), 23-34.
[30] Ghanem, N.; Yu, M.; Long, J.; Hatch, G.; Rubenstein, J. L. R.; Ekker, M., Distinct cisregulatory elements from the Dlx1/Dlx2 locus mark different progenitor cell populations in the ganglionic eminences and different subtypes of adult cortical interneurons. *Journal of Neuroscience,* **2007,** 27, (19), 5012-5022.
[31] Ghanem, N.; Jarinova, O.; Amores, A.; Long, Q.; Hatch, G.; Park, B. K.; Rubenstein, J. L. R.; Ekker, M., Regulatory roles of conserved intergenic domains in vertebrate *Dlx* bigene clusters. *Genome Res.,* **2003,** 13, 533-543.
[32] Powles, N.; Marshall, H.; Economou, A.; Chiang, C.; Murakami, A.; Dickson, C.; Krumlauf, R.; Maconochie, M., Regulatory analysis of the mouse Fgf3 gene: Control of embryonic expression patterns and dependence upon sonic hedgehog (Shh) signalling. *Dev. Dyn.,* **2004,** 230, (1), 44-56.
[33] Nishizaki, Y.; Shimazu, K.; Kondoh, H.; Sasaki, H., Identification of essential sequence motifs in the node/notochord enhancer of Foxa2 (Hnf3 beta) gene that are conserved across vertebrate species. *Mech. Dev.,* **2001,** 102, (1-2), 57-66.
[34] Sasaki, H.; Hogan, B. L. M., Enhancer analysis of the mouse HNF-3beta gene: regulatory elements for node/notchord and floor plate are independent and consist of mutiple sub-elements. *Genes Cells,* **1996,** 1, 59-72.
[35] Abbasi, A. A.; Paparidis, Z.; Malik, S.; Goode, D. K.; Callaway, H.; Elgar, G.; Grzeschik, K. H., Human GLI3 Intragenic Conserved Non-Coding Sequences Are Tissue-Specific Enhancers. *PLoS One,* **2007,** 2, (4).
[36] Drissen, R.; Guyot, B.; Zhang, L.; Atzberger, A.; Sloane-Stanley, J.; Wood, B.; Porcher, C.; Vyas, P., Lineage-specific combinatorial action of enhancers regulates mouse erythroid Gata1 expression. *Blood,* 115, (17), 3463-3471.
[37] Rojas, A.; De Val, S.; Heidt, A. B.; Xu, S. M.; Bristow, J.; Black, B. L., Gata4 expression in lateral mesoderm is downstream of BMP4 and is activated directly by Forkhead and GATA transcription factors through a distal enhancer element. *Development,* **2005,** 132, (15), 3405-3417.
[38] Schachterle, W.; Rojas, A.; Xu, S. M.; Black, B. L., ETS-dependent regulation of a distal Gata4 cardiac enhancer. *Dev. Biol.,* 361, (2), 439-449.
[39] Rojas, A.; Schachterle, W.; Xu, S. M.; Black, B. L., An Endoderm-Specific Transcriptional Enhancer from the Mouse Gata4 Gene Requires GATA and Homeodomain Protein-Binding Sites for Function In Vivo. *Dev. Dyn.,* **2009,** 238, (10), 2588-2598.
[40] Guittot, S. M.; Tetu, A.; Legault, E.; Pilon, N.; Silversides, D. W.; Viger, R. S., The proximal Gata4 promoter directs reporter gene expression to Sertoli cells during mouse gonadal development. *Biol. Reprod.,* **2007,** 76, (1), 85-95.
[41] Nakano, T.; Windrem, M.; Zappavigna, V.; Goldman, S. A., Identification of a conserved 125 base-pair Hb9 enhancer that specifies gene expression to spinal motor neurons. *Dev. Biol.,* **2005,** 283, (2), 474-485.
[42] Frasch, M.; Chen, X.; Lufkin, T., Evolutionary-conserved enhancers direct regionspecific expression of the murine Hoxa-1 and Hoxa-2 loci in both mice and Drosophila. *Development,* **1995,** 121, 957-974.
[43] Nonchev, S.; Vesque, C.; Maconochie, M.; Seitanidou, T.; Ariza-McNaughton, L.; Frain, M.; Marshall, H.; Sham, M. H.; Krumlauf, R.; Charnay, P., Segmental expression of Hoxa-2 in the hindbrain is directly regulated by Krox-20. *Development,* **1996,** 122, (2), 543-54.
[44] Tumpel, S.; Cambronero, F.; Sims, C.; Krumlauf, R.; Wiedemann, L. M., A regulatory module embedded in the coding region of Hoxa2 controls expression in rhombomere 2. *Proc. Natl. Acad. Sci. USA,* **2008,** 105, (51), 20077-20082.
[45] Manzanares, M.; Bel-Vialar, S.; Ariza-McNaughton, L.; Ferretti, E.; Marshall, H.; Maconochie, M. M.; Blasi, F.; Krumlauf, R., Independent regulation of initiation and maintenance phases of Hoxa3 expression in the vertebrate hindbrain involve auto- and crossregulatory mechanisms. *Development,* **2001,** 128, (18), 3595-3607.
[46] Knittel, T.; Kessel, M.; Kim, M. H.; Gruss, P., A conserved enhancer of the human and murine Hoxa-7 gene specifies the anterior boundary of expression during embryonal development. *Development,* **1995,** 121, (4), 1077-88.
[47] Langston, A. W.; Thompson, J. R.; Gudas, L. J., Retinoic acid-responsive enhancers located 3' of the Hox A and Hox B homeobox gene clusters. Functional analysis. *J. Biol. Chem.,* **1997,** 272, (4), 2167-75.
[48] Kwan, C. T.; Tsang, S. L.; Krumlauf, R.; Sham, M. H., Regulatory analysis of the mouse *Hoxb3* gene: multiple elements work in concert to direct temporal and spatial patterns of expression. *Dev. Biol.,* **2001,** 232, 176-190.
[49] Yau, T. O.; Kwan, C. T.; Jakt, L. M.; Stallwood, N.; Cordes, S.; Sham, M. H., Auto/cross-regulation in posterior Hindbrain of Hoxb3 expression and

spinal cord. *Dev. Biol.,* **2002,** 252, (2), 287-300.
[50] Whiting, J.; Marshall, H.; Cook, M.; Krumlauf, R.; Rigby, P. W. J.; Stott, D.; Allemann, R. K., Multiple spatially specific enhancers are required to reconstruct the pattern of hox-2.6 gene-expression. *Genes Dev.,* **1991,** 5, (11), 2048-2059.
[51] Gould, A.; Itasaki, N.; Krumlauf, R., Initiation of rhombomeric Hoxb4 expression requires induction by somites and a retinoid pathway. *Neuron,* **1998,** 21, (1), 39-51.
[52] Becker, D.; Jiang, Z.; Knodler, P.; Deinard, A. S.; Eid, R.; Kidd, K. K.; Shashikant, C. S.; Ruddle, F. H.; Schughart, K., Conserved regulatory element involved in the early onset of Hoxb6 gene expression. *Dev. Dyn.,* **1996,** 205, (1), 73-81.
[53] Shashikant, C. S.; Bieberich, C. J.; Belting, H. G.; Wang, J. C.; Borbely, M. A.; Ruddle, F. H., Regulation of Hoxc-8 during mouse embryonic development: identification and characterization of critical elements involved in early neural tube expression. *Development,* **1995,** 121, (12), 4339-47.
[54] Papenbrock, T.; Peterson, R. L.; Lee, R. S.; Hsu, T.; Kuroiwa, A.; Awgulewitsch, A., Murine Hoxc-9 gene contains a structurally and functionally conserved enhancer. *Dev. Dyn.,* **1998,** 212, 540-547.
[55] Popperl, H.; Featherstone, M. S., AN AUTOREGULATORY ELEMENT OF THE MURINE HOX-4.2 GENE. *EMBO J.,* **1992,** 11, (10), 3673-3680.
[56] Zhang, F.; Kovacs, E. N.; Featherstone, M. S., Murine Hoxd4 expression in the CNS requires multiple elements including a retinoic acid response element. *Mechanisms of Development,* **2000,** 96, (1), 79-89.
[57] MacKenzie, A.; Purdie, L.; Davidson, D.; Collinson, M.; Hill, R. E., Two enhancer domains control early aspects of the complex expression pattern of Msx1. *Mechanisms of Development,* **1997,** 62, (1), 29-40.
[58] Brugger, S. M.; Merrill, A. E.; Torres-Vazquez, J.; Wu, N.; Ting, M. C.; Cho, J. Y. M.; Dobias, S. L.; Yi, S. E.; Lyons, K.; Bei, J. R.; Arora, K.; Warrior, R.; Maxson, R., A phylogenetically conserved cis-regulatory module in the Msx2 promoter is sufficient for BMPdependent transcription in murine and Drosophila embryos. *Development,* **2004,** 131, (20), 5153-5165.
[59] Cheng, H. C.; Wang, C. K. L.; Upholt, W. B., Transcriptional regulation of Msx2 in the AERs of developing limbs is dependent on multiple closely spaced regulatory elements. *Dev.Biol.,* **2004,** 270, (2), 513-524.
[60] Summerbell, D.; Ashby, P. R.; Coutelle, O.; Cox, D.; Yee, S. P.; Rigby, P. W. J., The expression of Myf5 in the developing mouse embryo is controlled by discrete and dispersed enhancers specific for particular populations of skeletal muscle precursors. *Development,* **2000,** 127, (17), 3745-3757.
[61] Hadchouel, J.; Tajbakhsh, S.; Primig, M.; Chang, T. H. T.; Daubas, P.; Rocancourt, D.; Buckingham, M., Modular long-range regulation of Myf5 reveals unexpected heterogeneity between skeletal muscles in the mouse embryo. *Development,* **2000,** 127, (20), 4455-4467.
[62] Buchberger, A.; Nomokonova, N.; Arnold, H. H., Myf5 expression in somites and limb buds of mouse embryos is controlled by two distinct distal enhancer activities. *Development* **2003,** 130, (14), 3297-3307.
[63] Carvajal, J. J.; Cox, D.; Summerbell, D.; Rigby, P. W. J., A BAC transgenic analysis of the Mrf4/Myf5 locus reveals interdigitated elements that control activation and maintenance of gene expression during muscle development. *Development,* **2001,** 128, (10), 1857-1868.
[64] Fomin, M.; Nomokonova, N.; Arnold, H. H., Identification of a critical control element directing expression of the muscle-specific transcription factor MRF4 in the mouse embryo. *Dev. Biol.,* **2004,** 272, (2), 498-509.
[65] Chang, T. H. T.; Primig, M.; Hadchouel, J.; Tajbakhsh, S.; Rocancourt, D.; Fernandez, A.; Kappler, R.; Scherthan, H.; Buckingham, M., An enhancer directs differential expression of the linked Mrf4 and Myf5 myogenic regulatory genes in the mouse. *Dev. Biol.,* **2004,** 269, (2), 595-608.
[66] Reecy, J. M.; Li, X. Y.; Yamada, M.; DeMayo, F. J.; Newman, C. S.; Harvey, R. P.; Schwartz, R. J., Identification of upstream regulatory regions in the heart-expressed homeobox gene Nkx2-5. *Development,* **1999,** 126, (4), 839-849.
[67] Adachi, H.; Saijoh, Y.; Mochida, K.; Ohishi, S.; Hashiguchi, H.; Hirao, A.; Hamada, H., Determination of left right asymmetric expression of nodal by a left side-specific enhancer with sequence similarity to a lefty-2 enhancer. *Genes Dev.,* **1999,** 13, (12), 1589-1600.
[68] Saijoh, Y.; Oki, S.; Tanaka, C.; Nakamura, T.; Adachi, H.; Yan, Y. T.; Shen, M. M.; Hamada, H., Two nodal-responsive enhancers control left-right asymmetric expression of Nodal. *Dev. Dyn.,* **2005,** 232, (4), 1031-1036.
[69] Okazawa, H.; Okamoto, K.; Ishino, F.; Ishinokaneko, T.; Takeda, S.; Toyoda, Y.; Muramatsu, M.; Hamada, H., THE OCT3 GENE, A GENE FOR AN EMBRYONIC TRANSCRIPTION FACTOR, IS CONTROLLED BY A RETINOIC ACID REPRESSIBLE ENHANCER. *EMBO J.,* **1991,** 10, (10), 2997-3005.
[70] Kimura, C.; Takeda, N.; Suzuki, M.; Oshimura, M.; Aizawa, S.; Matsuo, I., Cis-acting elements conserved between mouse and pufferfish Otx2 genes govern the expression in mesencephalic neural crest cells. *Development,* **1997,** 124, (20), 3929-3941.
[71] Kurokawa, D.; Kiyonari, H.; Nakayama, R.; Kimura-Yoshida, C.; Matsuo, I.; Aizawa, S., Regulation of Otx2 expression and its functions in mouse forebrain and midbrain. *Development,* **2004,** 131, (14), 3319-3331.
[72] Kammandel, B.; Chowdhury, K.; Stoykova, A.; Aparicio, S.; Brenner, S.; Gruss, P., Distinct *cis*-essential modules direct the time-space pattern of the *Pax6* gene activity. *Dev. Biol.,* **1999,** 205, 79-97.
[73] Xu, Z. P.; Saunders, G. F., PAX6 intronic sequence targets expression to the spinal cord. *Dev. Genet.,* **1998,** 23, (4), 259-63.
[74] Xu, P. X.; Zhang, X.; Heaney, S.; Yoon, A.; Michelson, A. M.; Maas, R. L., Regulation of Pax6 expression is conserved between mice and flies. *Development,* **1999,** 126, (2), 383-95.
[75] Kleinjan, D.; Seawright, A.; Childs, A.; van Heyningen, V., Conserved elements in Pax6 intron 7 involved in (auto)regulation and alternative transcription. *Dev. Biol.,* **2004,** 265, 462-477.
[76] Sato, S.; Ikeda, K.; Shioi, G.; Ochi, H.; Ogino, H.; Yajima, H.; Kawakami, K., Conserved expression of mouse Six1 in the pre-placodal region (PPR) and identification of an enhancer for the rostral PPR. *Dev. Biol.,* **2010,** 344, (1), 158-171.
[77] Goke, J.; Jung, M.; Behrens, S.; Chavez, L.; O'Keeffe, S.; Timmermann, B.; Lehrach, H.; Adjaye, J.; Vingron, M., Combinatorial Binding in Human and Mouse Embryonic Stem Cells Identifies Conserved Enhancers Active in Early Embryonic Development. *PLoS Comput. Biol.,* 7, (12).
[78] Antonellis, A.; Huynh, J. L.; Lee-Lin, S. Q.; Vinton, R. M.; Renaud, G.; Loftus, S. K.; Elliot, G.; Wolfsberg, T. G.; Green, E. D.; McCallion, A. S.; Pavan, W. J., Identification of Neural Crest and Glial Enhancers at the Mouse Sox10 Locus through Transgenesis in Zebrafish. *PLoS Genet.,* **2008,** 4, (9).
